# Predictors of Alcohol Consumption Among Younger Adults During the First Phase of the COVID-19 Pandemic

**DOI:** 10.3389/fpsyt.2021.748158

**Published:** 2021-10-12

**Authors:** Lasse Brandt, Ricarda Evens, Simon Reiche, Roman M. Marek, Daa Un Moon, Elisa Groß, Amy Romanello, Dario Jalilzadeh Masah, Matteo Scicchitano, Stefan Gutwinski, Christiane Montag, Tomislav Majić, Inge Mick

**Affiliations:** ^1^Department of Psychiatry and Psychotherapy, Charité Campus Mitte, Charité –Universitätsmedizin Berlin, Corporate Member of Freie Universität Berlin, Humboldt-Universität zu Berlin, and Berlin Institute of Health, Berlin, Germany; ^2^Berlin-Brandenburg Academy of Sciences and Humanities, Berlin, Germany; ^3^German Rheumatism Research Centre Berlin, Berlin, Germany; ^4^Humboldt-Universität zu Berlin, Berlin School of Mind and Brain, Berlin, Germany

**Keywords:** alcohol, COVID-19, pandemic, quarantine, predictor, younger adults

## Abstract

**Background:** The COVID-19 pandemic may lead to negative mental health effects but the effect on alcohol consumption among younger adults is unclear. We assess predictors of change in alcohol consumption during the first phase of the COVID-19 pandemic among younger adults.

**Methods:** This cross-sectional internet-based survey was part of an overarching project, the Corona Drug Survey, which was conducted from April 30 to August 4, 2020. Participants of any sex and ≥18 years old were included. The primary outcome measure was change in alcohol consumption during the early COVID-19 pandemic. We implemented an ordinal logistic regression to assess the effect (odds ratio [OR] and 95% confidence interval [CI]) of the following predictors: quarantine restrictions on leaving the residence, number of individuals in the household, problematic alcohol consumption before the pandemic (CAGE [cutting down, annoyance by criticism, guilty feeling, and eye-opener] score), personal concern regarding the pandemic, age, and sex.

**Results:** 3,321 participants with a mean age of 32 (SD: 13) years were included in this study. 70.4% of participants reported less or unchanged alcohol consumption in the recent 4 weeks of the pandemic compared to before the pandemic. A higher number of individuals in the household was associated with a reduced alcohol consumption (OR = 0.869; 95% CI = 0.815–0.927). No quarantine restrictions on leaving the residence (OR = 1.593; 95% CI = 1.397–1.817), a higher age (1.006; 1.001-1.011), and female sex (compared to males: 1.206; 1.062–1.371) were associated with an increase in alcohol consumption. The CAGE score before the pandemic (OR = 0.983; 95% CI = 0.931–1.037) and the pandemic concern (0.927; 0.857–1.003) were not associated with a significant change in alcohol consumption. Celebrations were no longer frequent drinking occasions during the pandemic compared to before the pandemic. The majority of participants (60.9%) did not use alcohol drinking as a coping mechanism to mitigate negative effects of the pandemic.

**Interpretation:** In this cohort of younger adults with fewer celebratory drinking occasions, restrictions on leaving the residence and the number of persons in the household were the strongest predictors of reduced alcohol consumption during the early phase of the pandemic.

## Introduction

Alcohol consumption is highly prevalent in many societies worldwide and alcohol-related health disorders are associated with a substantial personal and social impact (1). In the United States, according to the National Survey on Drug Use and Health (NSDUH) in 2019, 69.5% of participants reported alcohol consumption in the past year (2). Alcohol was estimated to be the third leading preventable cause of death (3, 4).

Currently, an unprecedented number of people is exposed to the ongoing corona virus disease (COVID-19) pandemic (5) and a large proportion of the general population is affected socially and economically by the pandemic and infection control measures (e.g., quarantine) (6, 7): for instance, disruptions of social networks may be associated with gathering and movement restrictions and economic uncertainties may be related to restructuring or loss of work.

COVID-19 has been categorized as a disease with priority for research in public health by the World Health Organization (8). Previous reports suggested increased mental health adverse effects such as depression, anxiety, stress-related disorders, and anger related to the pandemic and infection control measures (6, 7). However, the effect of the current pandemic on alcohol consumption in younger adults has not yet been well-established, as studies that specifically addressed alcohol consumption during the COVID-19 pandemic reported heterogeneous results that ranged from increased or unchanged to decreased alcohol consumption (9–19).

In a recent study by Ritter et al. (Drug Policy Modelling Program, Australia) (13), the authors suggested that the change in alcohol consumption was determined by individual sociodemographic predictors: for example, the lack of licensed venues serving alcohol during quarantine was strongly associated with a decreased alcohol consumption in younger adults (18–24 years), while older adults were less affected by closure of licensed venues. Based on findings from a European online survey with 40,064 participants in 21 countries (including Germany), Manthey et al. argued that the reduction in drinking occasions during the pandemic led to an average decrease in alcohol consumption (19). However, Koopmann et al. reported that subjects with higher levels of perceived stress during quarantine measures consumed more alcohol since the beginning of quarantine measures (online survey with 2,102 participants from the German general population) (10). These inconsistent findings illustrate the need to establish the impact of specific predictors on changes in alcohol consumption during a global public health crisis like the COVID-19 pandemic.

The living situation (e.g., number of individuals in the household) may be of particular importance for individuals that are experiencing quarantine restrictions on leaving the residence as stress during quarantine may be related to social isolation on the one hand, and interpersonal conflicts on the other. Furthermore, a series of other factors such as the pattern of alcohol consumption prior to lockdown, age, concern related to the pandemic, and age of onset on alcohol consumption may have an effect on alcohol consumption (13, 17).

The aim of this study is to investigate the effects of (a) quarantine restrictions on leaving the residence, (b) number of individuals in the household, (c) personal concern regarding the pandemic, (d) problematic alcohol consumption before the pandemic, (e) age, and (f) sex on changes in alcohol consumption during the early stages of the pandemic. For this purpose, we implemented a cross-sectional internet-based survey to recruit an international sample of individuals.

## Methods

This study was part of an overarching project: the Corona Drug Survey (CDS; https://corona-drugsurvey.org/; now defunct). It was approved by The Ethics Committee of the Charité – Universitätsmedizin Berlin, Germany. The aim of CDS was to assess the motivations, settings, and patterns of use of different groups of psychoactive substances during the early COVID-19 pandemic. This article focuses on alcohol consumption, whereas reports on other substances will be published accordingly. The study was designed as a cross-sectional internet-based survey and it was conducted in the early phase of the pandemic (from April 30 to August 4, 2020). Participants remained anonymous and could answer the questionnaire in five languages: English, German, Spanish, Italian, and Korean. Translations were performed by native speakers. Adult participants (≥18 years old and no upper age limit) of any sex that were fluent in any of the five languages were included. Only participants with a previous consumption of at least one of the following substances during the time of 2019–2020 were included: alcohol, nicotine, cannabis, benzodiazepines, cocaine, amphetamine, methamphetamine, ecstasy, serotonergic psychedelics, dissociatives, opioids, gamma hydroxybutyrate/butyrolactone (GHB/GBL), and new psychoactive substances. Participants were recruited via digital postings on platforms such as facebook.com and reddit.com, via e-mail announcements, and via articles in online magazines such as vice.com (i.e., convenience sampling). The postings included links to the survey, which was hosted by the secure web-based platform SoSci Survey [soscisurvey.de (20)].

### Primary Outcome Measure

The primary outcome measure was a self-reported change in alcohol consumption during the early COVID-19 pandemic. Participants answered the following question: “Has the amount of your alcohol consumption changed in the last 4 weeks compared to before the corona crisis?”. Change in alcohol consumption was categorized according to a five-level Likert scale: 1 = much less, 2 = slightly less, 3 = unchanged, 4 = slightly more, and 5 = much more.

### Secondary Outcome Measure

The secondary outcome measure was mean frequency of alcohol consumption. The mean frequency of alcohol consumption was defined according to a three-tier classification: 1 = none, 2 = less than daily, and 3 = daily. Participants were asked to score the mean frequency of alcohol consumption during two time periods: before the pandemic and during the recent 4 weeks of the pandemic.

### Predictors

Restrictions on leaving the residence (on the day of the survey: yes, no), number of individuals in the household (0, 1, 2–3, >3 individuals), problematic features of alcohol consumption [CAGE score (21)], personal concerns related to the development of the COVID-19 pandemic (four-tier item), age (years), and sex (female, male, other) were included as predictors.

Problematic features of alcohol consumption were assessed with the clinical screening instrument “CAGE questionnaire” (21): CAGE is an acronym derived from four questions related to problematic features of alcohol consumption (cutting down, annoyance by criticism, guilty feeling, and eye-opener).

Personal concerns related to the development of the COVID-19 pandemic (i.e., “pandemic concern”) were assessed with a four-tier item: 1 = no concern, 2 = little concern, 3 = much concern, and 4 = very much concern.

### Cohort Characteristics

Cohort characteristics were reported descriptively and included demographic and social parameters (such as country of origin, level of education, work and living situation), patterns and motivations of alcohol use, and items regarding pandemic exposure (such as testing positive for the Severe Acute Respiratory Syndrome Corona Virus 2 [SARS-CoV-2]). Items are reported in the Supplement 1.

### Statistics

An ordinal logistic regression was implemented to assess the effect of predictors on the primary outcome parameter (change in alcohol consumption during the early COVID-19 pandemic) and to estimate the odds ratio (OR) and 95% confidence interval (CI) (22).

Restrictions on leaving the residence, number of individuals in the household, pandemic concern, CAGE score, age, and sex were included as independent variables in the ordinal logistic regression, whereas change in alcohol consumption during the early COVID-19 pandemic was the dependent variable. For the analysis of the secondary outcome parameter, we assessed the difference in frequency of alcohol consumption between the two time periods with a Wilcoxon signed-rank test. Other cohort characteristics are reported descriptively. In this exploratory analysis, *p* values <0.05 are considered to be statistically significant and all *p* values are reported with four decimals. We report mean ± standard deviation. Regarding group characteristics, percentage (%) of participants out of the total sample is reported in the main manuscript and the number (*n*) of participants out of the total sample is reported in the Supplement 1. Percentages may not sum to 100 due to rounding. SPSS (version 23, IBM) was used for statistical analysis.

## Results

### Sample Characteristics

In total, *N* = 3,321 individuals from the overarching CDS project replied to the primary question regarding change in frequency of alcohol consumption and were included in this analysis (Supplement 1). The included participants chose to answer the questionnaire in the following languages: Spanish (34.1%), German (27.7%), English (26.4%), Italian (6.2%), and Korean (5.7%).

The most frequently reported locations were Germany (29.7%), Mexico (11.6%), Colombia (7.7%), Argentina (7.6%), India (5.3%), Italy (5.0%), South Africa (3.3%), United Kingdom (2.6%), United States of America (2.0%), Bolivia (1.9%), and Chile (1.3%). Hence, the majority of participants reported locations in Germany and Latin America. The sex distribution was 52.3% male, 45.7% female, and 1.2% other. The mean age was 32 ± 13 (min. 18, max. 92) years.

The cohort was well-educated and reported the following highest degrees: university diploma, bachelor's degree, or master's degree (46.1%); high school diploma, A-levels, or secondary school leaving qualification (26.3%); vocational training or technical apprenticeship (8.1%); General Educational Development examination (GED) or alternative school leaving certificate (6.6%); technical college entrance qualification (5.8%); doctorate (3.6%); no graduation (3.1%); or secondary school leaving certificate (0.4%).

Before the pandemic, subjects had the following occupations: permanent employment for an unlimited period (31.2%), student employment (16.0%), self-employment (14.6%), permanent employment for a limited period (11.0%), seeking work (10.3%), freelancer (5.3%), temporary employment (4.1%), retired (3.4%), unpaid domestic or family work (“housewife / househusband”) (3.0%), or parental leave (0.7%). After the beginning of the pandemic, 45.0% of individuals experienced a change in their occupation: reduction of working hours/furlough (21.3%), livelihood in jeopardy (12.9%), loss of job (12.5%), or workplace endangered by pandemic (11.9%).

Participants were mostly not living alone and shared their household with the following number of individuals: 2–3 individuals (39.1%), >3 individuals (27.5%), 1 individual (18.4%), no other individual (15.0%).

Children were not living in most households of the participants: no children (73.6%), 1 child (14.1%), 2–3 children (11.4%), and >3 children (0.9%).

In 2019–2020, participants had consumed the following substances: alcohol (100%), cannabinoids (65.3%), nicotine (60.5%), cocaine (32.5%), MDMA/ecstasy (28.7%), psychedelics such as LSD or psilocybin (28.3%), amphetamines (21.1%), dissociatives such as ketamine (16.1%), benzodiazepines (16.1%), opioids (7%), new psychoactive substances such as mephedrone (4.5%), and GHB / GBL (2.9%).

### Pandemic-Related Restrictions and Impact on Personal Lives

On the day of the survey, 97.2% of participants reported experiencing at least one of the following quarantine-related social measures: restricted social contacts (88.6%), schools / kindergarten closed (72.5%), most shops closed (46.1%), restrictions on leaving the residence (38.3%), and other measures (21.3%). Only a very small fraction of participants (0.2%) had ever been tested positive for the Severe Acute Respiratory Syndrome Corona Virus 2 (SARS-CoV-2).

During the recent four weeks of the pandemic individuals had varying degrees of concern related to the development of the pandemic: no concern (11.1%), little concern (48.1%), much concern (30.2%), and very much concern (10.6%).

The most frequently reported concerns were economical, followed by health and social concerns. Specifically, the participants experienced the following pandemic-related concerns: “there could be an economic crisis” (67.5%), “I or many others could lose their jobs and income” (63.7%), “I or my family/friends could become sick with coronavirus” (60.2%), “the healthcare system is overburdened” (50.4%), “it could cause me or many others psychological problems” (41.2%), “there is no capacity for the treatment of other diseases” (36.3%), “there could be a political crisis” (36.3%), “international conflicts could intensify” (33.1%), “I or many people do not have enough social contact or connection” (32.7%), “I or many others could die in a very short time” (23.7%), “there could be difficulties in affording or acquiring substances (e.g., alcohol, cannabis, cocaine, etc.)” (21.7%), “I am treated to my disadvantage in case of hospital treatment” (10.7%), and “I could suffer from symptoms of withdrawal” (7.4%).

Of note, 66.9% of participants also reported positive effects of quarantine measures. Positive effects consisted of the following: more spare time (36.9%), relief from obligations (29.9%), more contact with partner, family, or friends (27.6%), new hobbies (22.5%), and new freedoms (14.5%).

### Patterns and Motivations of Alcohol Use

**Figure 1:**
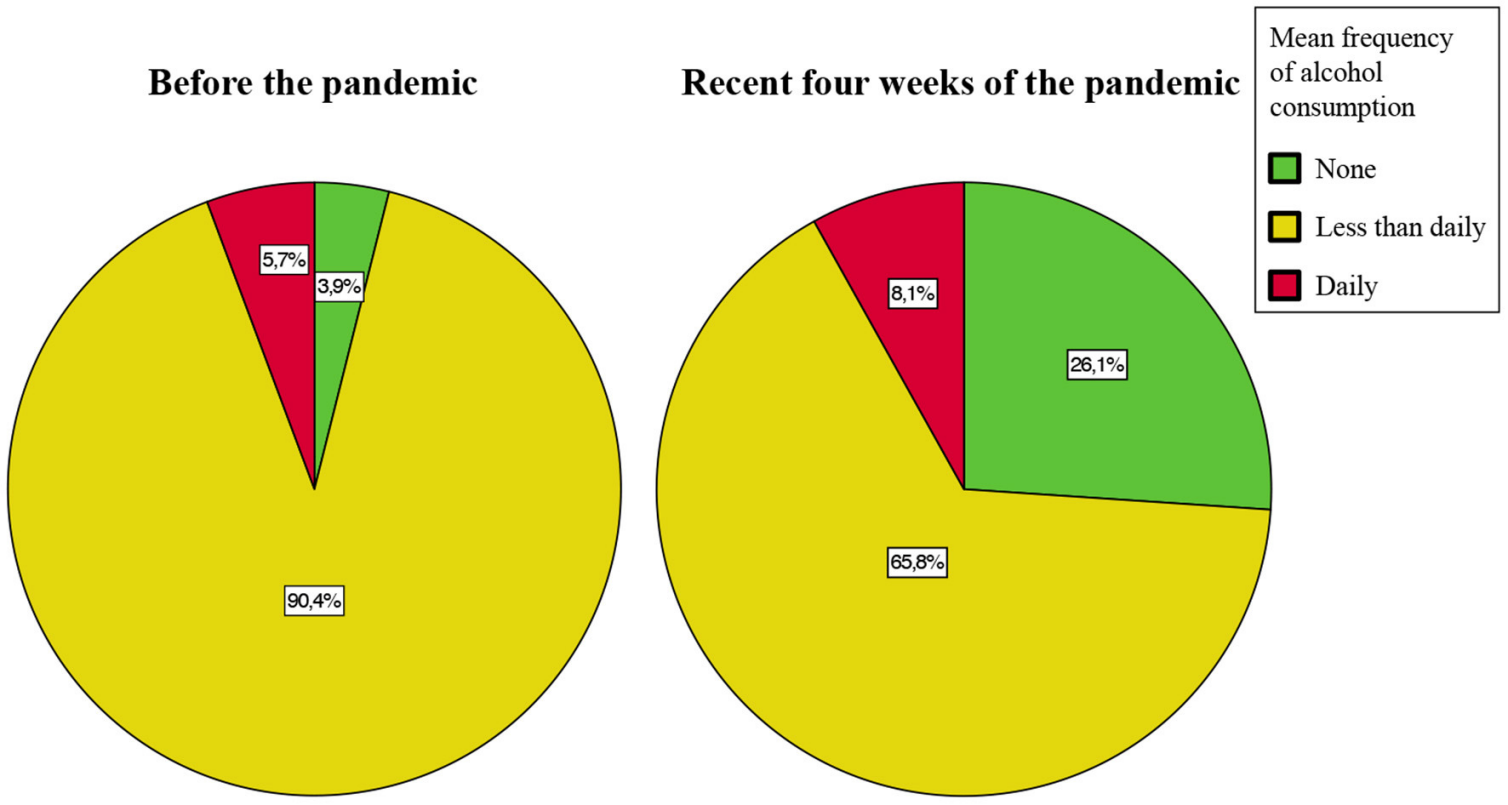
Mean frequency of alcohol consumption during the early COVID-19 pandemic. The pie charts illustrate the mean frequency of alcohol consumption before the pandemic (left) and during the recent 4 weeks of the pandemic (right). Compared to before pandemic, the mean frequency of alcohol consumption decreased during the recent 4 weeks of pandemic. The percentages indicate the proportion of individuals from the sample excl. *n* = 2 individuals did not report mean frequency of alcohol consumption, i.e., this figure includes data from *n* = 3,319 individuals. Absolute number are reported in the Supplement 1.

Compared to before pandemic, the mean frequency of alcohol consumption decreased during the recent 4 weeks of the pandemic (Figure 1): 24.7% (*n* = 820) of participants showed a decrease in alcohol consumption, whereas only 5.3% (*n* = 175) of participants showed an increase in alcohol consumption (alcohol consumption: 1 = none, 2 = less than daily, 3 = daily; alcohol consumption before the pandemic vs. recent 4 weeks of the pandemic = 2 vs. 1.8; *z* = −20.22, *p* < 0.0001, Wilcoxon signed-rank test).

Before the pandemic, participants reported alcohol consumption of at least 4–5 alcoholic drinks within few hours (based on definition of “binge-drinking” according to the National Institute on Alcohol Abuse and Alcoholism – NIAAA^1^) mostly at weekends: weekends (50.9%), never (27.2%), weekdays (19.0%), or daily (2.6%). During the recent four weeks of the pandemic, 50.6% of participants did not change, 33.5% of participants reported less frequent, and 15.2% reported more frequent “binge-drinking.”

Before the pandemic, participants had varying degrees of CAGE scores: 0 (32.1%), 1 (23.0%), 2 (21.1%), 3 (16.3%), and 4 (2.8%). The majority of participants (60.9%) did not use alcohol to cope with distress from the pandemic. Among the reasons for alcohol consumption, celebrations decreased most during the recent 4 weeks of the pandemic compared to before the pandemic: before the pandemic, reasons for alcohol consumption included “to celebrate” (72.1%), “for pleasure” (71.6%), “for relaxation” (46.6%), “because friends/family used alcohol” (43.4%), and “out of boredom” (27.1%). During the recent four weeks of the pandemic, reported reasons for alcohol consumption included “for pleasure” (49.9%), “for relaxation” (38.0%), “out of boredom” (26.3%), “to celebrate” (24.4%), and “because friends/family used alcohol” (21.8%). Before the pandemic, participants had consumed the following types of alcoholic drinks: beer (70.2%), wine and sparkling wine (48.5%), liquor (38.1%), liqueurs (15.1%), and alcopops (e.g., flavored malt beverage) (11.2%). During the recent four weeks of the pandemic, the majority of participants (84.1%) did not change the type of alcoholic drink, while 8.3% of participants consumed alcoholic drinks with less alcohol content and 7.4% of participants consumed alcoholic drinks with higher alcohol content.

### Predictors of Change in Alcohol Consumption During the Early COVID-19 Pandemic

The majority of participants (70.4%) reported less or unchanged alcohol consumption during the early COVID-19 pandemic: much less (28.2%), slightly less (14.5%), unchanged (27.7%), slightly more (22.1%), or much more (7.4%).

We assessed the effect of the predictors quarantine restrictions on leaving the residence, number of individuals in the household, pandemic concern, CAGE score, age, and sex on change in alcohol consumption during the early COVID-19 pandemic (Table 1):

a higher number of individuals in the household was associated with a reduced alcohol consumption (OR = 0.869; 95% CI = 0.815–0.927; Table 1).

**Table 1:** Predictors of change in alcohol consumption during the early COVID-19 pandemic.

**Predictor**	**Reference**	**Wald chi-square**	**OR**	**95% CI**	***P***
No quarantine restrictions on leaving the residence	Quarantine restrictions on leaving the residence	48.141	1.593	1.397–1.817	<0.0001
Number of individuals in household	One unit (number) of individuals in household less	18.482	0.869	0.815–0.927	<0.0001
Pandemic concern	One unit (score) of pandemic concern less	3.563	0.927	0.857–1.003	0.0591
CAGE score	One unit (score) of CAGE score less	0.409	0.983	0.931–1.037	0.5225
Age	One unit (years) of age less	6.430	1.006	1.001–1.011	0.0112
Female sex	Male sex	8.316	1.206	1.062–1.371	0.0039
Other sex	Male sex	0.053	0.936	0.532–1.647	0.8180

*OR, odds ratio; CI, confidence interval. The degree of freedom (df) was 1 for each parameter. The variables included in the ordinal logistic regression analysis met the assumptions of the statistical model (Supplement 2)*.

No quarantine restrictions on leaving the residence was associated with an increased alcohol consumption (OR = 1.593; 95% CI = 1.397–1.817; Table 1).

The CAGE score before the pandemic (OR = 0.983; 95% CI = 0.931–1.037; Table 1) and the level of pandemic concern (OR = 0.927; 95% CI = 0.857–1.003; Table 1) were not associated with a significant change in alcohol consumption.

A higher age was associated with an increase in alcohol consumption (OR = 1.006; 95% CI = 1.001–1.011; Table 1). Compared with males, females were associated with increased alcohol consumption (OR = 1.206; 95% CI = 1.062–1.371; Table 1).

## Discussion

This study aimed to assess predictors of change in alcohol consumption during the early stages of the COVID-19 pandemic. In this cohort of younger adults (32 ± 13 years, min. 18, max. 92), the majority (70.4%) of participants reported less or unchanged alcohol consumption. Quarantine restrictions on leaving the residence and the number of individuals in the household were the strongest predictors of reduced alcohol consumption during the early phase of the pandemic. A younger age and male sex were also associated with reduced alcohol consumption. In this cohort, the CAGE score before the pandemic and the pandemic concern were not associated with a significant change in alcohol consumption during the early phase of the pandemic.

The majority of participants (60.9%) did not use alcohol to cope with distress from the pandemic. In the time period before the pandemic, celebration was a drinking occasion for 72.1% of individuals, in contrast to only 24.4% of individuals during the recent 4 weeks of the pandemic. The change in motivation for alcohol consumption may be related to the majority (97.2%) of participants experiencing at least one pandemic-related social restriction.

The majority of the cohort was well-educated and lived with other adults. It is possible that participants may have implemented other, functional coping mechanisms not related to alcohol consumption, such as social interactions with individuals from the same household. Younger adults may also have had fewer drinking occasions when they were restricted to the residence with family members. The effect appeared pronounced for younger males compared to females.

A recent publication by Kilian et al. is in agreement with our findings and confirms a decreased alcohol consumption during the first months of the COVID-19 pandemic (23). The authors also implemented a cross-sectional on-line survey in a similar timeframe (April 24 to July 22, 2020) as our study. They included data from 31,964 individuals from twenty-one European countries. They report that decreases in alcohol consumption were mainly driven by a reduced frequency of “heavy episodic consumption events.” Similarly, our data indicated a reduced frequency of “binge-drinking” in 33.5% of participants. Another recent study by Garcia-Cerde et al. included 33 countries and two territories from Latin America and the Caribbean and demonstrated that the prevalence of alcohol use reduced from 77.5% in 2019 to 65% during the pandemic (24). These findings suggest that the reduction in alcohol consumption among younger adults may have occurred in different geographical locations and cultural settings.

In a study by Callinan et al. analyzing 1,684 Australians aged 18–65 years who drank at least monthly, results showed a decrease in harmful drinking during social distancing measures (16). The authors attributed their findings to the closure of licensed premises and social distancing measures (16). Based on a European online survey in 21 countries with 40,064 participants, Manthey et al. showed a mean decrease in alcohol consumption since the pandemic started (19). Manthey and colleagues suggested that the mean decrease in alcohol consumption was mostly related to a reduction in drinking occasions (19). Our analysis confirmed a reduction in celebratory drinking occasions during the early phase of the pandemic, which suggests that quarantine measures such as restrictions on leaving the residence could be predictors of reduced alcohol consumption in younger adults.

In a representative survey of more than 2,000 people commissioned by Alcohol Change UK, 35% of individuals reduced or stopped drinking alcohol after the lockdown began (11). Here, the report suggested an awareness among a proportion of the participants that lockdown measures could be a risk for increased alcohol consumption: 38% of participants reported to take active steps to manage their drinking (11). However, other reports suggested increases in alcohol consumption since the beginning of the pandemic (10, 12). Ritter et al. concluded that—while many people (2/3) appeared to be resilient and continued to perform self-care—there was not a simple pattern of changes in alcohol consumption during the pandemic, thus research should further investigate home drinking settings (13). It is important to note that the findings of our study do not imply that the risk of alcohol use disorders—defined according to standard diagnostic criteria by the Diagnostic and Statistical Manual of Mental Disorders (DSM) (25) or the International Statistical Classification of Diseases and Related Health Problems (ICD) (26)—is reduced during the early COVID-19 pandemic and it is strongly recommended to maintain access to substance addiction services and mental health care services, especially for vulnerable populations at risk for adverse mental health effects during the pandemic such as individuals with a history of mental disorders (6, 7, 27).

### Strengths and Limitations

This cross-sectional internet-based survey was implemented in the first phase of the pandemic and a large sample of younger adults from different countries was recruited. The design of the study allowed for participation regardless of restrictions of movement or interaction during the pandemic, provided that internet access was functioning.

However, the study was limited in several aspects: first, the study had a cross-sectional design and inferences regarding the causalities of the findings are limited. Second, generalizability was limited due to convenience sampling. Third, the self-reported outcomes may potentially be confounded by social desirability and recall bias (28). Fourth, the participants were mainly located in Germany and Latin America and sample characteristics such as cultural features may be heterogeneous in our sample. Furthermore, generalizability of the results from this study are limited for locations beyond Germany and Latin America. For example, results may differ significantly in populations with other social conventions regarding alcohol consumption or different implementation of quarantine measures.

Fifth, an assessment of mental health disorders according to standardized diagnostic criteria was not implemented in this anonymous internet-based survey, which constitutes a limitation of this study. An assessment of mental health disorders in future studies would increase validity of findings.

Sixth, the survey did not specifically target individuals with an alcohol use disorder (AUD). Findings may differ for individuals with AUD and changes in risk of relapse during the pandemic was not addressed in this study.

Moreover, while the majority of the cohort was younger, well-educated, and living with other adult individuals, individuals with less social and economic resources may potentially be more strongly affected by adverse effects of the pandemic and quarantine measures. Individuals with lower educational levels were underrepresented in this sample. Seventh, a proportion of participants also consumed other substances and it could by hypothesized that a reduction in alcohol consumption may be compensated by an increased consumption of other substances. However, also the consumption of cannabinoids was not found to be increased in our analysis of data from the corona drug survey (data on cannabinoids will be published accordingly).

Eighth, the psychosocial impact of the pandemic may change as the pandemic develops. Thus, the effects of the pandemic exposure on alcohol consumption may change during the development of pandemic, which would not be reflected in our cross-sectional study.

## Conclusion

Our findings indicate that quarantine restrictions on leaving the residence and a higher number of individuals in the household were associated with reduced alcohol consumption in younger adults during the early phase of the pandemic. Fewer drinking occasions and a paucity of alcohol drinking as coping mechanism may have contributed to reduced alcohol consumption.

## Data Availability Statement

The original contributions presented in the study are included in the article/Supplementary Material, further inquiries can be directed to the corresponding author.

## Ethics Statement

The studies involving human participants were reviewed and approved by the Ethics Committee of the Charité—Universitätsmedizin Berlin, Germany. The participants provided their written informed consent to participate in this study.

## Author Contributions

IM, RE, SR, CM, SG, and TM: concept and design. LB and IM: statistical analysis. All authors: drafting of the protocol, acquisition, analysis, or interpretation of data and critical revision of the protocol for important intellectual content.

## Conflict of Interest

The authors declare that the research was conducted in the absence of any commercial or financial relationships that could be construed as a potential conflict of interest.

## Publisher's Note

All claims expressed in this article are solely those of the authors and do not necessarily represent those of their affiliated organizations, or those of the publisher, the editors and the reviewers. Any product that may be evaluated in this article, or claim that may be made by its manufacturer, is not guaranteed or endorsed by the publisher.
