# Supplementary material for: Predictors of Alcohol Consumption Among Younger Adults During the First Phase of the COVID-19 Pandemic
Source: Front Psychiatry. 2021 Oct 12;12:748158. doi: 10.3389/fpsyt.2021.748158 (PMC8546114; doi:10.3389/fpsyt.2021.748158)
Supplement: Supplementary file 1 [file Table_1.pdf]

Supplementary Material

Table Supplement 1. Absolute values and percentages for each variable.

**Variables**	**Number (n) of participants out of total sample (N=3,321)**	**Percentage (%) of participants out of total sample (N=3,321)**
**Language**		
**English**	876	26.4
**German**	920	27.7
**Italian**	205	6.2
**Korean**	188	5.7
**Spanish**	1132	34.1
No response	0	0
**Location**		
**Germany**	986	29.7
**Mexico**	386	11.6
**Colombia**	255	7.7
**Argentina**	253	7.6
**USA**	65	2.0
**India**	176	5.3
**United Kingdom**	87	2.6
**Italy**	167	5.0
**Bolivia**	62	1.9
**Korea**	5	0.2

**Austria**	5	0.2
**Ecuador**	28	0.8
**Chile**	42	1.3
**South Africa**	108	3.3
**Other**	696	21.0
No response	0	0
**Sex**		
**Female**	1517	45.7
**Other**	40	1.2
**Male**	1736	52.3
No response	28	0.8
**Age**		
**Years: mean 32 (standard deviation 13)**	3316	99.8
No response	5	0.2
**Degree**		
**No graduation**	103	3.1
**Secondary school leaving certificate**	14	0.4
**General Educational Development examination (GED) or alternative school leaving certificate**	220	6.6
**Technical college entrance qualification**	192	5.8
**High school diploma, A-levels, or secondary school leaving qualification**	873	26.3
**University diploma, bachelor's degree, or master's degree**	1532	46.1
**Doctorate**	119	3.6
**Vocational training or technical apprenticeship**	268	8.1
No response	0	0

**Occupation before pandemic**		
**Seeking work**	343	10.3
**Unpaid domestic or family work (housewife/househusband)**	99	3.0
**Parental leave**	22	0.7
**Student employment**	531	16.0
**Temporary employment**	136	4.1
**Permanent employment for a limited period**	365	11.0
**Permanent employment for an unlimited period**	1037	31.2
**Freelancer**	175	5.3
**Self-employment**	485	14.6
**Retired**	114	3.4
No response	14	0.4
**Change in occupation after beginning of the pandemic**		
**Experienced a change in their occupation**	1494	45.0
**Loss of job**	416	12.5
**Reduction of working hours / furlough**	706	21.3
**Workplace endangered by pandemic**	395	11.9
**Livelihood in jeopardy**	428	12.9
No response	0	0
**Number of other individuals in the household**		
**Alone**	497	15.0
**One**	611	18.4
**Two to three**	1298	39.1
**More than three**	913	27.5

No response	2	0.1
**Children in the household**		
**None**	2443	73.6
**One**	467	14.1
**Two to three**	379	11.4
**More than three**	30	0.9
No response	2	0.1
**Participants consumed the following substances in 2019-2020**		
**Alcohol**	3321	100.0
**Cannabinoids**	2170	65.3
**Nicotine**	2009	60.5
**Cocaine**	1080	32.5
**MDMA/ecstasy**	952	28.7
**Psychedelics**	939	28.3
**Amphetamines**	701	21.1
**Dissociatives**	534	16.1
**Benzodiazepines**	534	16.1
**Opioids**	231	7.0
**New psychoactive substances**	151	4.5
**GHB/GBL**	96	2.9
No response	0	0
**Quarantine-related social measures**		
**None**	92	2.8
**Restricted social contacts**	2949	88.8
**Schools/kindergarten closed**	2408	72.5

**Most shops closed**	1530	46.1
**Restrictions on leaving the residence**	1271	38.3
**Other measures**	707	21.3
No response	0	0
**Ever been tested positive for the Severe Acute Respiratory Syndrome Corona Virus 2 (SARS-CoV-2)**		
**Yes**	8	0.2
No response	2	0.1
**Degree of concern related to the development of the pandemic**		
**No concern**	369	11.1
**Little concern**	1599	48.1
**Much concern**	1002	30.2
**Very much concern**	351	10.6
No response	0	0
**Specific concerns related to the development of the pandemic**		
**"I or my family/friends could become sick with coronavirus"**	1999	60.2
**"I or many others could die in a very short time"**	787	23.7
**"The healthcare system is overburdened"**	1674	50.4
**"There is no capacity for the treatment of other diseases"**	1204	36.3
**"I or many people do not have enough social contact or connection"**	1087	32.7
**"It could cause me or many others psychological problems"**	1367	41.2
**"There could be an economic crisis"**	2241	67.5
**"There could be a political crisis"**	1205	36.3

**"International conflicts could intensify"**	1100	33.1
**"I or many others could lose their jobs and income"**	2115	63.7
**"There could be difficulties in affording or acquiring substances (e.g. alcohol, cannabis, cocaine, etc.)"**	721	21.7
**"I could suffer from symptoms of withdrawal"**	245	7.4
**"I am treated to my disadvantage in case of hospital treatment"**	356	10.7
No response	0	0
**Mean frequency of alcohol consumption: before the pandemic**		
**None**	130	3.9
**Less than daily**	3000	90.3
**Daily**	189	5.7
**No response**	2	0.1
**Mean frequency of alcohol consumption: recent four weeks of the pandemic**		
**None**	865	26.0
**Less than daily**	2185	65.8
**Daily**	269	8.1
No response	2	0.1
**Positive effects of quarantine measures**		
**Yes**	2222	66.9
**Relief from obligations**	993	29.9
**New freedoms**	481	14.5
**More contact with partner, family, or friends**	915	27.6
**More spare time**	1226	36.9

**New hobbies**	747	22.5
No response	0	0
**Consumption of the following types of alcoholic drinks before the pandemic**	373	
**Alcopops**	373	11.2
**Beer**	2331	70.2
**Wine and sparkling wine**	1610	48.5
**Liquor**	1265	38.1
**Liqueurs**	503	15.1
No response	0	0
**Change of the type of alcoholic drink during the recent four weeks of the pandemic**		
**Less alcohol content**	277	8.3
**Higher alcohol content**	247	7.4
**No change**	2793	84.1
No response	4	0.1
**Use of alcohol to cope with distress from the pandemic**		
**Not at all true**	2024	60.9
**Slightly true**	572	17.2
**Moderately true**	298	9.0
**Very true**	119	3.6
**Absolutely true**	49	1.5
No response	22	0.7
**Before the pandemic: alcohol consumption of at least 4-5 alcoholic drinks within few hours**		
**Weekends**	1692	50.9
**Never**	902	27.2

**Weekdays**	632	19.0
**Daily**	87	2.6
No response	8	0.2
**Recent four weeks of the pandemic: change in alcohol consumption of at least 4-5 alcoholic drinks within few hours**		
**No change**	1681	50.6
**More frequent alcohol consumption of at least 4-5 alcoholic drinks within few hours**	506	15.2
**Less frequent alcohol consumption of at least 4-5 alcoholic drinks within few hours**	1113	33.5
No response	21	0.6
**CAGE score**		
**0**	1067	32.1
**1**	765	23.0
**2**	700	21.1
**3**	541	16.3
**4**	93	2.8
No response	155	4.7
**Before the pandemic: reasons for alcohol consumption**		
**To celebrate**	2395	72.1
**Out of boredom**	899	27.1
**For pleasure**	2379	71.6
**For relaxation**	1547	46.6
**Because friends/family used alcohol**	1441	43.4
No response	155	4.7

**Recent four weeks of the pandemic: reasons for alcohol consumption**		
**To celebrate**	809	24.4
**Out of boredom**	872	26.3
**For pleasure**	1657	49.9
**For relaxation**	1261	38.0
**Because friends/family used alcohol**	724	21.8
No response	155	4.7
**Change of alcohol consumption in the recent four weeks compared to before the COVID-19 pandemic**		
**Much less**	937	28.2
**Slightly less**	482	14.5
**Unchanged**	921	27.7
**Slightly more**	734	22.1
**Much more**	247	7.4
No response	0	0

Supplement 2. Assumptions of the ordinal logistic regression analysis

The variables included in the ordinal logistic regression analysis met the assumptions of the statistical model (1): the dependent variable (i.e. change in alcohol consumption) was treated as an ordinal variable and the independent variables were treated as continuous variables (i.e. age, number of individuals in the household, personal concern regarding the pandemic, and problematic alcohol consumption before the pandemic) or categorical variables (i.e. sex and quarantine restrictions on leaving the residence). The assumption of no multicollinearity was assessed by examining the Variation Inflation Factors (VIFs) and the result (VIFs <1.1) indicated no multicollinearity (Table 1) (2). The assumption of proportional odds was assessed with a full likelihood ratio test and the result (p >.05) indicated proportional odds (Table 2) (3).

Table 1. Assumption of no multicollinearity

Predictor	Reference	Tolerance	Variation Inflation Factor (VIF)
**No quarantine restrictions on leaving the residence**	Quarantine restrictions on leaving the residence	0.971	1.050
**Number of individuals in household**	One unit (number) of individuals in household less	0.944	1.060
**Pandemic concern**	One unit (score) of pandemic concern less	0.952	1.050
**CAGE score**	One unit (score) of CAGE score less	0.967	1.034
**Age**	One unit (years) of age less	0.919	1.088
**Female sex**	Male sex	0.972	1.029
**Other sex**	Male sex	0.983	1.017

Table 2. Assumption of proportional odds

Test	Chi-square	Degrees of freedom	p
**Full likelihood ratio test**	31.724	21	0.0624

References for Supplement 2

1. Bender R, Grouven U. Ordinal logistic regression in medical research. J Roy Coll Phys Lond (1997) 31:546–51.

2. Dormann CF, Elith J, Bacher S, Buchmann C, Carl G, Carré G, Marquéz JRG, Gruber B, Lafourcade B, Leitão PJ, et al. Collinearity: a review of methods to deal with it and a simulation study evaluating their performance. Ecography (2013) 36:27–46.

3. Brant R. Assessing Proportionality in the Proportional Odds Model for Ordinal Logistic Regression. Biometrics (1990) 46:1171–1178.
